# The complete mitogenome of *Argentina brasiliensis* Kobyliansky, 2004 and a phylogenetic analyses of the order Argentiniformes

**DOI:** 10.1590/1678-4685-GMB-2024-0170

**Published:** 2024-12-06

**Authors:** Claudio Oliveira, Heloísa De Cia Caixeta, Marcelo Roberto Souto de Melo

**Affiliations:** ¹ Universidade Estadual Paulista ‘Júlio de Mesquita Filho’, Instituto de Biociências, São Paulo, SP, Brazil.; ² Universidade de São Paulo, Instituto Oceanográfico, São Paulo, SP, Brazil.

**Keywords:** mtDNA, biodiversity, systematic, fish, deep-sea fauna

## Abstract

The deep sea environment is the largest environment and host some of the most extreme ecosystems on Earth, therefore, possessing a large and unique fish diversity that encompasses about 15% of all known species. Our knowledge about these fishes is still very limited in many biological fields basically due to the complexity to obtain specimens for research. In the present study, we describe the complete mitochondrial genome of *Argentina brasiliensis*, aiming a species characterization and the study of the phylogenetic relationships in the order Argentiniformes. The mitogenome is composed by 13 protein-coding genes, 2 rRNA genes, 22 tRNA genes, and a control region (D-loop), as found in other vertebrates. The phylogenetic results show that the order Argentiniformes is composed by two family groups the first formed by Argentinidae and Opisthoproctidae and the second formed by Bathylagidae and Microstomatidae. Additionally, we found that the genus *Argentina* is not monophyletic, and we suggest additional studies in the family Argentinidae to better investigate this question.

The order Argentiniformes, commonly known as marine smelts, includes small-sized, silver or dark, bathypelagic or benthopelagic ocean fishes (Nelson et al., 2016). The order comprises 99 valid species classified into the following four families: Argentinidae (2 genera and 29 species), Opisthoproctidae (10 genera and 23 species), Microstomatidae (3 genera and 22 species), and Bathylagidae (8 genera and 25 species) (Van der Laan and Fricke, 2024). The hypotheses about the phylogenetic position of Argentiniformes have changed, since some early studies suggested a close relationship with Alepocephaliformes (see Nelson *et al*., 2016 for a complete discussion) but recent studies suggest that they are more closely related to Salmoniformes (see a revision in Near and Thacker, 2024). On the other hand, morphological and molecular evidence agree that the order is monophyletic (Near and Thacker, 2024). 

Although the deep sea is the largest and one of the most extreme environment on Earth, it still remains largely unexplored mainly due the high-cost of the cruises, technological restrictions, research funding, and availability of research vessels (Bell et al., 2023). During the DEEP-OCEAN project, two oceanographic cruises were conducted aboard the R/V *Alpha Crucis* to collect deep-sea fish between 250 and 1,520 m on the continental slope of Southern and Southeastern Brazil, resulting in the obtention of about 120 Actinopterygii species, including the samples of *Argentina brasiliensis* that are studied here. *Argentina brasiliensis* was described by Kobyliansky (2004) based on specimens collected off the southern Brazilian coast and, although locally abundant (MRSM person. obs.), it is apparently endemic to that region. 

The specimen of *Argentina brasiliensis* analyzed in the present study was captured onboard the Brazilian RV *Alpha Crucis* using bottom trawling, on the Brazilian continental slope, S 28°11’613” W 47°12’301”, in 27/03/2022, and preserved in the collection of the Laboratório de Biologia e Genética de Peixes (LBP) do Instituto de Biociências da Universidade Estadual Paulista under the catalog number of LBP 31774-102017. Collection permits were issued by the Instituto Chico Mendes de Conservação da Biodiversidade (SISBIO #28054-4, 82624-1), Secretaria da Comissão Interministerial para Recursos do Mar da Marinha do Brasil (Portaria No. 223), and the Comitê de Ética em Uso de Animais em Pesquisa e Ensino do Instituto Oceanográfico da Universidade de São Paulo (CEUA #16 to MRSM). Soon after the collection, the specimens were photographed, had a tissue sample removed for molecular analysis preserved in 96 GL ethanol, and the voucher was fixed in 10% formaldehyde and preserved in 70% ethanol, for identification conference. 

Total genomic DNA from muscle tissue was extracted using the Wizard Genomic DNA Purification kit (Promega Corp., WI, USA). Library preparation and sequencing were done by Arbor Biosciences (Ann Arbor, MI). Sequencing was performed on the Illumina NovaSeq S4 platform at Arbor Biosciences. The sequencing generated 6,465,268 pair reads. After sequencing, adapter contamination, low quality bases and the sequences containing ambiguous base calls were trimmed using the Illumiprocessor (github.com/faircloth-lab/illumiprocessor) within Phyluce (Faircloth, 2016). After trimming, Illumina reads were assembled into contigs using the program SPAdes 3.13.1 (Prjibelski et al., 2020). Contigs were analyzed in the program Geneious Primer (Geneious 2024.0.4, https://www.geneious.com) where the complete mitochondrial genome was identified by the molecule size. The mitogenome structure and gene identification was done in the webserver MitoFish (http:// mitofish.aori.u-tokyo.ac.jp/) (Zhu et al., 2023). The complete mitochondrial genome sequence (mtDNA) of *A. brasiliensis* was deposited in NCBI (GenBank no. PQ032107).

To construct a phylogenetic tree, the amino acids of the 13 protein coding genes of *A. brasiliensis* were aligned with the genes of all species listed in Figure 1 using the program Muscle 5.1 in Geneious Primer (Geneious 2024.0.4, https://www.geneious.com). The best substitution model was selected in ModelFinder (Kalyaanamoorthy et al., 2017) in the program IQ-Tree 2 (Minh et al., 2020) in the webserver Cipres (Miller et al., 2010). The maximum likelihood (ML) tree was constructed in the program IQ-Tree 2 (Minh *et al*., 2020) and tested using 1000 bootstrap pseudoreplications. The phylogenetic tree was visualized and edited using FigTree v. 1.4.4 (http://tree.bio.ed.ac.uk/software/figtree/).

**Figure 1-:**
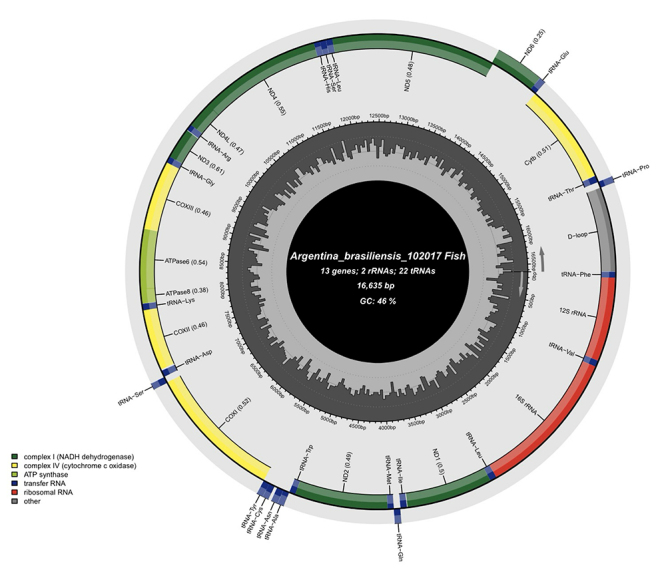
Gene map of the mitochondrial genome of Argentina brasiliensis. The genome contained two rRNA genes (in red), 13 coding genes (in green), 22 tRNA genes (in blue), and a control region (D-loop) (in gray).

SPAdes analyses exploring K values from 21 to 77 recovered from 443 to 2863 perfect loops. The mtDNA of *A. brasiliensis* is a closed circular molecule of 16,635 bp in size (Figure 1), which is like those of not only teleost species but also terrestrial vertebrates. It possesses the typical combination of 37 genes, including 13 protein-coding genes, 22 tRNA genes, and two genes for ribosomal RNA subunits (12S rRNA and 16S rRNA). Twenty-eight genes were located on the heavy (H) strand, while the ND6 gene and eight tRNA genes were transcribed from the light (L) strand (Figure 1). Like other animal mitochondrial genomes, *A. brasiliensis* contained no introns and possessed two non-coding regions, an OL and a control region or displacement loop (D-loop) region. The OL region, consisting of 32 nucleotides, was situated between tRNA_Asn_ and tRNA_Cys_ and was oriented on the L-strand in a cluster of five tRNA genes (WANCY region). D-loop region of *A. brasiliensis* has 956 nucleotides which represented 5.7 % of the total mitogenome. 

A ML analyses generated a well-supported tree with four lineages, representing different families (Figure 2). The only group identified as non-monophyletic was the genus *Argentina* since the species *Glossanodon semisfasciatus* was positioned among species of *Argentina* (Figure 2). 

**Figure 2-:**
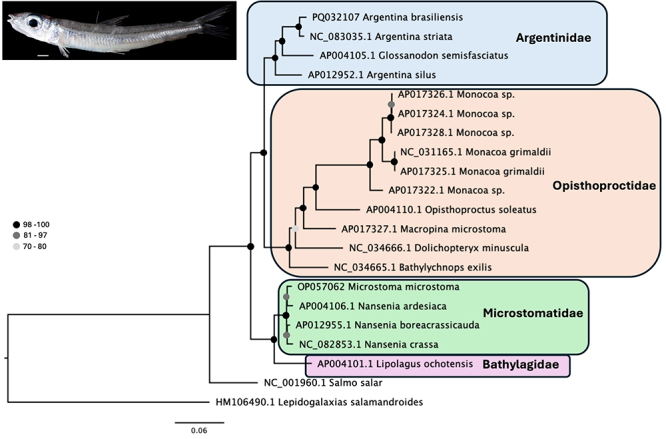
Maximum Likelihood tree based on amino acid sequences from 13 mitochondrial genes. Circles show the node bootstrap support values. GenBank numbers are presented before species names. Above, on the left, a photo of a specimen of *Argentina brasiliensis*.

The mitochondrial genome of *Argentina brasiliensis* shows the common mitochondrial gene order found in fishes (Ishiguro et al., 2003) as well as in most vertebrates (Poulsen et al., 2013). This gene order was also found in its congeners, *A. silus* (Poulsen, 2015) and *A. striata* (GenBank number NC_083035), as well as in all other argentiniforms (Ishiguro *et al*. 2003; present study). 

The family Argentinidae includes only two genera, *Argentina* and *Glossanodon* (Van der Laan and Fricke, 2024). The mtDNA sequence of *G. semisfasciatus* was determined by Ishiguro et al. (2003) in a study about basal euteleostean relationships but not previously compared with other Argentiniformes. The present study was the first evaluate the phylogenetic relationship among *G. semisfasciatus* and species of *Argentina* and our results show that *G. semisfasciatus* belongs to the family Argentinidae, but its taxonomic status needs to be revised since it was observed that it is positioned nested among the species of *Argentina*, thus turning the genus paraphyletic (Figure 2). 

Both morphological and molecular evidence supports that the order Argentiniformes is monophyletic (review in Near and Thacker, 2024), however the relationship among families is still controversial. Using morphological characters, Rosen (1974) first found Argentinidae as sister group of Bathylagidae plus Opisthoproctidae and the same result was found in posterior morphological studies by Begle (1992) and Patterson and Johnson (1997). Previous molecular studies, involving a single representative species of each family, found Argentinidae as sister group of Opisthoproctidae, and Bathylagidae as sister groups of Microstomatidae (Li et al., 2010; Near *et al*., 2012). The result found by Betancur-R et al. (2017), including five species, was different since they found Bathylagidae as sister group of all remained families, followed by Microstomatidae as sister group of Argentinidae plus Opisthoproctidae. Our results, including 16 species, corroborate the studies of Li *et al*. (2010) and Near *et al*. (2012), showing that the order Argentiniformes has two family groups, the first including Argentinidae and Opisthoproctidae and the second including Bathylagidae and Microstomatidae (Figure 2). This result expands significatively our knowledge about Argentiniformes and open opportunities for further studies in the group. 
